# Supplementary material for: A high-resolution mRNA expression time course of embryonic development in zebrafish
Source: eLife. 2017 Nov 16;6:e30860. doi: 10.7554/eLife.30860 (PMC5690287; doi:10.7554/eLife.30860)
Supplement: Supplementary file 6. [file elife-30860-supp6.zip › biolayout-clusters-files/Cluster086-genes.html]

Cluster086


# Cluster086: Genes

| Ensembl ID | Gene Name | Chr | Start | End | Biotype |
| --- | --- | --- | --- | --- | --- |
| ENSDARG00000041490 | afp4 | 16 | 45937437 | 45943334 | protein\_coding |
| ENSDARG00000045980 | cst14b.1 | 5 | 4081428 | 4091680 | protein\_coding |
| ENSDARG00000039173 | ctslb | 12 | 16495264 | 16497883 | protein\_coding |
| ENSDARG00000074306 | ctslb.1 | 12 | 16569710 | 16572218 | protein\_coding |
| ENSDARG00000101089 | gart | 18 | 160820 | 188582 | protein\_coding |
| ENSDARG00000019122 | he1b | 22 | 14068583 | 14074622 | protein\_coding |
| ENSDARG00000095254 | si:dkey-239j18.2 | 12 | 16470534 | 16639370 | protein\_coding |
| ENSDARG00000089806 | si:dkey-239j18.3 | 12 | 16512568 | 16514753 | protein\_coding |
| ENSDARG00000095744 | si:dkey-269i1.4 | 12 | 16752771 | 16755262 | protein\_coding |
| ENSDARG00000100833 | si:dkey-269i1.4.1 | 20 | 12559246 | 12563583 | protein\_coding |
| ENSDARG00000089750 | si:dkey-26g8.5 | 12 | 16797537 | 16801288 | protein\_coding |
| ENSDARG00000101407 | tgm1l4 | 23 | 46240647 | 46250912 | protein\_coding |
| ENSDARG00000101364 | wfikkn1 | 1 | 58863071 | 58865313 | protein\_coding |
| ENSDARG00000094559 | zgc:174855 | 12 | 16470701 | 16819445 | protein\_coding |
